# Rational Engineering and Preparation of Horseradish Peroxidase C

**DOI:** 10.3390/molecules31203730

**Published:** 2026-10-17

**Authors:** Juran Cui, Qifeng Yu, Sen Yang, Quanxi Wang, Yaping Wang, Ben Rao

**Affiliations:** 1 National Biopesticide Engineering Technology Research Center, Hubei Biopesticide Engineering Research Center, Hubei Academy of Agricultural Sciences, Biopesticide Branch of Hubei Innovation Centre of Agricultural Science and Technology, Wuhan 430064, China; cjrhubu@163.com (J.C.); yqfhubu@163.com (Q.Y.);; 2 State Key Laboratory of Biocatalysis and Enzyme, Engineering Hubei Collaborative Innovation Center for Green Transformation of Bio-Resources, Hubei Key Laboratory of Industrial Biotechnology, College of Life Sciences of Hubei University, Hubei University, Wuhan 430062, China

**Keywords:** horseradish peroxidase, *Pichia pastoris*, protein engineering, glycosylation modification, high-density fermentation, lyophilized formulation

## Abstract

To address the persistent challenges of low yield and significant batch-to-batch variability inherent in the traditional plant-based extraction of horseradish peroxidase (HRP), this study aimed to develop an efficient production strategy for HRP-C through rational molecular design and process optimization. A recombinant engineering strain was constructed in *Pichia pastoris* GS115, and site-directed mutagenesis was performed based on FoldX virtual screening to evaluate mutations affecting enzyme stability and catalytic properties. A comprehensive 50 L scale process was established, integrating high-density fermentation, purification, and lyophilized formulation. Results indicated that exogenous supplementation with 5 μM hemin significantly enhanced enzymatic activity. The HRP-C-D282I variant exhibited a 17.7% increase in specific activity and a 2.14-fold extension of half-life at 60 °C compared with the wild-type enzyme. Furthermore, Endo H-mediated deglycosylation was associated with a dose-dependent decrease in HRP-C-D282I activity, suggesting that *N*-glycosylation contributes to enzyme performance under the conditions tested. The optimized process achieved a recombinant protein concentration of 216 mg/L during fermentation and a purified enzyme specific activity of 2600 U/mg, with a lyophilized powder reconstitution time of less than 60 s. This study provides a proof-of-concept basis for the potential industrial production and application of recombinant HRP.

## 1. Introduction

Horseradish peroxidase (HRP, EC 1.11.1.7), a heme-containing glycoprotein originally isolated from the roots of *Armoracia rusticana*, is one of the most extensively utilized enzymes in biotechnology, clinical diagnostics, and environmental remediation [1,2]. Its prominence stems from high catalytic efficiency, broad substrate affinity, remarkable stability, and excellent water solubility. Among its isoforms, HRP-C is the most abundant and well-characterized, consisting of a 308-amino acid polypeptide chain with a molecular weight of approximately 34 kDa and a carbohydrate content of approximately 18%, primarily composed of mannose and *N*-acetylglucosamine linked via *N*-glycosidic bonds at Asn13, Asn57, and Asn255 [3,4]. The enzyme’s stable three-dimensional structure is maintained by a heme prosthetic group coordinated with His170, two structural Ca^2+^ ions, and four conserved disulfide bonds [5,6].

The catalytic cycle of HRP follows the classic Chance–George mechanism, proceeding through highly reactive intermediates (Compounds I and II) to oxidize a wide array of reducing substrates—including guaiacol and 2,2′-azino-bis(3-ethylbenzothiazoline-6-sulfonic acid) (ABTS)—using hydrogen peroxide as an oxidant [7,8]. This versatile catalytic capability underpins its widespread applications, ranging from biosensors and immunoassays such as enzyme-linked immunosorbent assay (ELISA) and immunohistochemistry (IHC) to bioremediation of phenolic pollutants and food safety testing [9,10,11]. In synthetic biology, HRP has expanded beyond its traditional role as a labeling enzyme, serving as a critical component in CRISPR-Cas-based nucleic acid detection and the synthesis of conductive polymers and biomedical hydrogels [12,13,14,15].

Despite its industrial significance, the traditional plant extraction of HRP faces critical limitations, including low yields, high batch-to-batch variability, and seasonal supply constraints [16]. Recombinant expression offers a viable alternative, yet challenges persist across different host systems. While *Escherichia coli* (*E. coli*) lacks the machinery for proper folding and glycosylation of this complex enzyme, leading to inactive inclusion bodies [17], eukaryotic systems like *Pichia pastoris* (*P. pastoris*) and *Saccharomyces cerevisiae* offer distinct advantages. *P. pastoris*, in particular, combines a powerful methanol-inducible promoter (AOX1), an optimized fed-batch fermentation system, and advanced eukaryotic post-translational modification systems, making it the preferred host for scalable HRP production [18,19]. Recent advances, such as the development of the SynPic-X platform, further enhance its safety and productivity by replacing methanol induction with alternative carbon sources [20].

However, achieving high-level expression of functionally active HRP in *P. pastoris* requires addressing several bottlenecks. Heme incorporation is often a limiting step in HRP expression. Therefore, exogenous hemin supplementation was optimized. Furthermore, the stability and substrate specificity of native HRP often fall short of the demanding requirements of industrial applications, such as operation under extreme pH, high temperatures, or in the presence of metal ions. Protein engineering, through rational design and directed evolution, provides powerful strategies to overcome these limitations by tailoring the enzyme’s structure–function relationship [21]. Previous studies have demonstrated that site-directed mutagenesis is an effective approach for improving HRP catalytic performance and stability. Humer and Spadiut enhanced HRP properties through rational amino acid substitutions, while Kim et al. engineered HRP-C variants with improved resistance to radical-induced inactivation [22,23]. Additionally, glycosylation plays a pivotal yet complex role in modulating HRP’s stability, solubility, and catalytic efficiency, requiring careful characterization and control in recombinant systems [24].

To address these challenges, this study aimed to develop a scalable and efficient production platform for HRP-C in *P. pastoris*. The specific objectives were to: (1) optimize recombinant expression and purification processes, including the critical supplementation of hemin; (2) employ rational protein design to engineer variants with enhanced catalytic activity, thermostability, and modified substrate specificity; (3) elucidate the impact of *N*-glycosylation on enzyme structure and function; and (4) establish a stabilized lyophilized formulation for long-term storage. This work provides a proof-of-concept framework toward potential scalable and industrial HRP production, and further multi-batch pilot-scale validations are needed before real industrial deployment.

## 2. Results and Discussion

### 2.1. Enhancement of Active HRP-C Production by Hemin Supplementation

The catalytic activity of horseradish peroxidase depends on the incorporation of the heme prosthetic group, which is essential for electron transfer during substrate oxidation. Although *P. pastoris* provides an efficient platform for recombinant protein production, insufficient heme availability may limit the formation of catalytically active HRP-C. Therefore, hemin supplementation was investigated as an approach to improve the production of active recombinant HRP-C.

**Figure 1:**
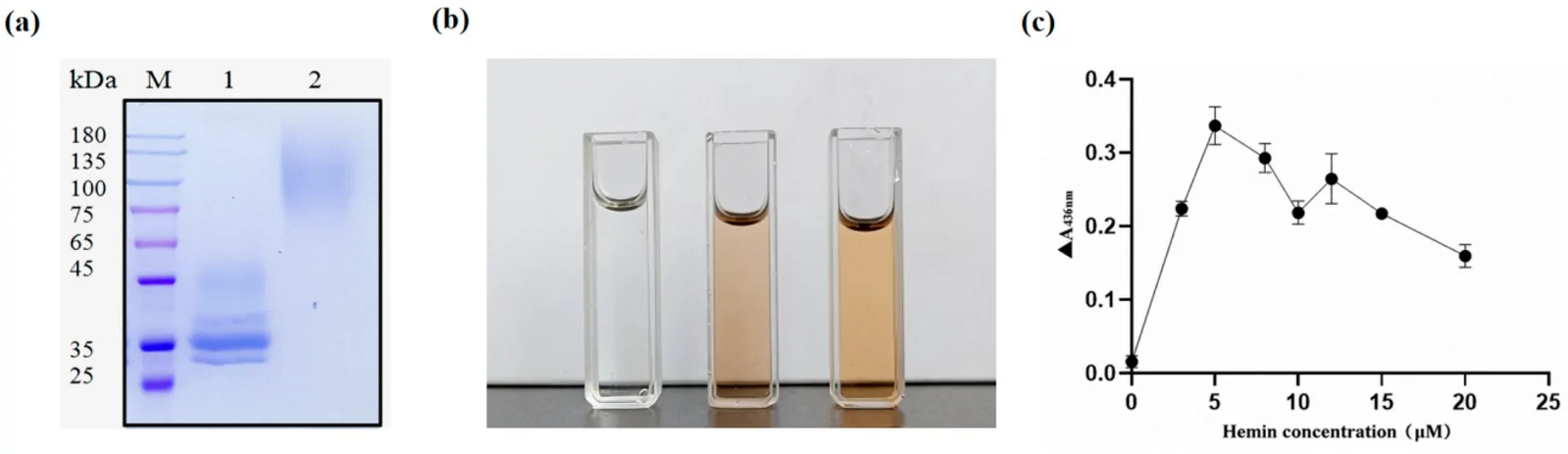
Recombinant expression and hemin-assisted activity optimization of HRP-C. (**a**) BDM-HRP-C Shaking Bottle SDS-PAGE Gel Electrophoresis, M: Protein pre-stained marker; lane 1: Endo H-treated deglycosylated HRP-C from culture supernatant; lane 2: HRP-C protein from culture supernatant. (**b**) The guaiacol–H_2_O_2_ chromogenic reaction. The left vial represents the blank control, while the middle and right vials show the HRP-C-catalyzed chromogenic reactions. (**c**) Effects of different hemin concentrations on HRP-C enzyme activity. ΔA_436nm_represents the absorbance change at 436 nm during the reaction. All experiments were performed in biological triplicate (n = 3), and data are presented as mean ± standard deviation (SD)..

Different concentrations of hemin were supplemented during the expression process, and the effects on HRP-C production were evaluated through protein expression analysis and enzymatic activity determination. Recombinant HRP-C expression was successfully detected after induction, while supplementation with hemin promoted the formation of active enzyme compared with the control group (Figure 1a,b). Further optimization showed that the addition of 5 μM hemin resulted in the highest peroxidase activity, indicating that an appropriate heme supply was beneficial for improving the catalytic efficiency of recombinant HRP-C (Figure 1c).

The improvement in enzyme activity after hemin supplementation suggested that heme availability represents an important limiting factor during recombinant HRP-C production. Increasing the supply of heme precursors may facilitate the combination of apoprotein and prosthetic group, thereby increasing the proportion of correctly folded and catalytically active enzyme molecules. Therefore, 5 μM hemin supplementation was selected as the optimal condition for subsequent HRP-C engineering and characterization.

### 2.2. Rational Design and Screening of HRP-C Variants

Although hemin supplementation improved the production of active HRP-C, further enhancement of enzyme performance required modification of the intrinsic properties of the protein. Therefore, a rational engineering strategy combining sequence conservation analysis, structural analysis, and computational prediction was applied to identify potential beneficial mutation sites.

**Figure 2:**
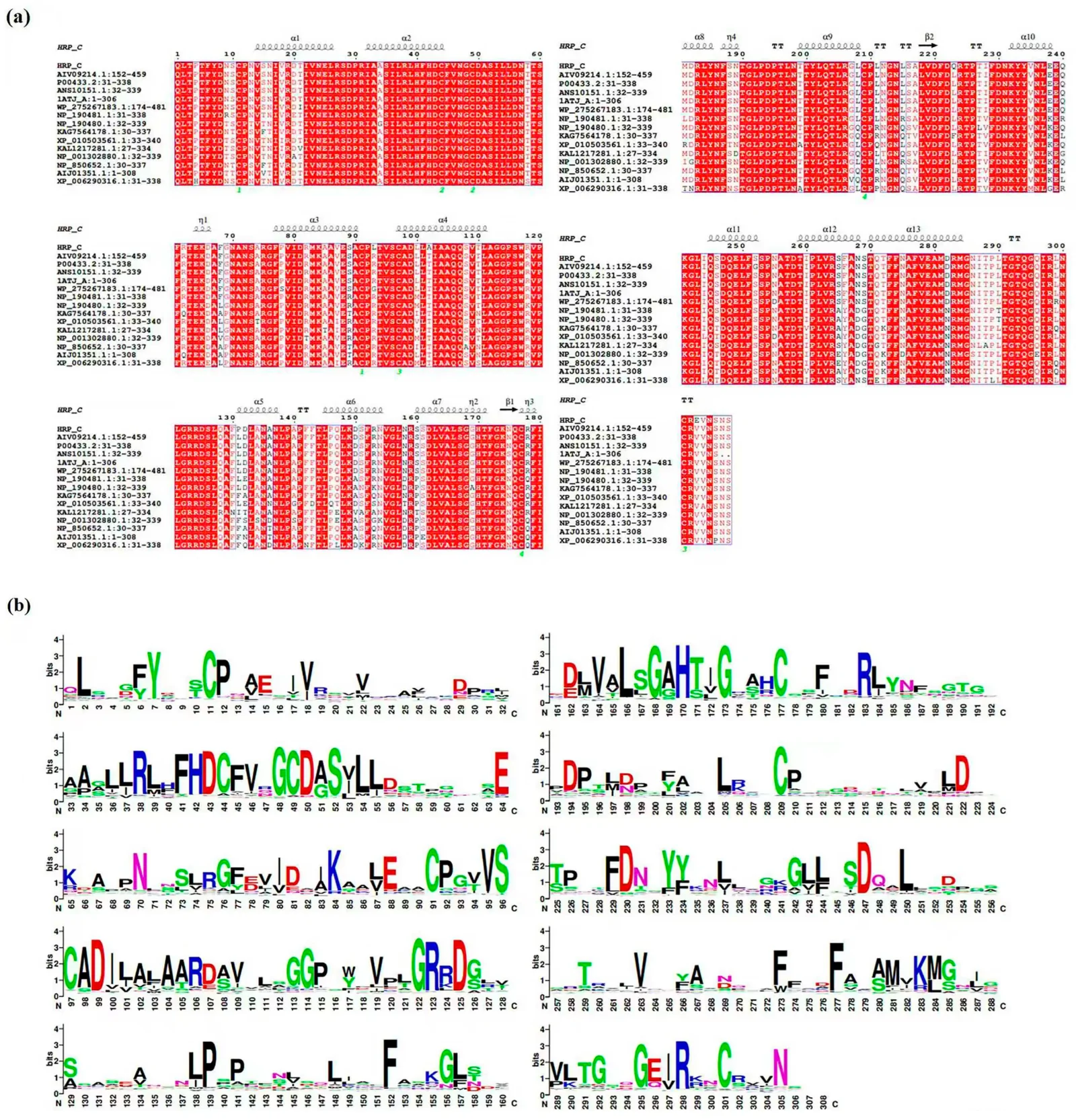
Multiple Sequence Alignment and Conservation Analysis of Peroxidase. (**a**) Sequence conservation diagram based on Clustal Omega (v1.2.4) alignment. (**b**) Sequence conservation profile generated by WebLogo (v3.7.1). The Y-axis represents information content (bits), and the X-axis represents the amino acid sequence position (1–308). The height of the stacked characters reflects the frequency of occurrence of each amino acid at that position, and colors denote different amino acid types.

Multiple sequence alignment of HRP homologs revealed highly conserved regions among different plant peroxidases, while several variable residues were identified as potential targets for engineering. Conservation analysis further identified surface-exposed and non-conserved amino acid residues that may influence enzyme stability and catalytic performance (Figure 2a,b). Based on these results, candidate residues were selected for computational evaluation and subsequent experimental verification.

**Figure 3:**
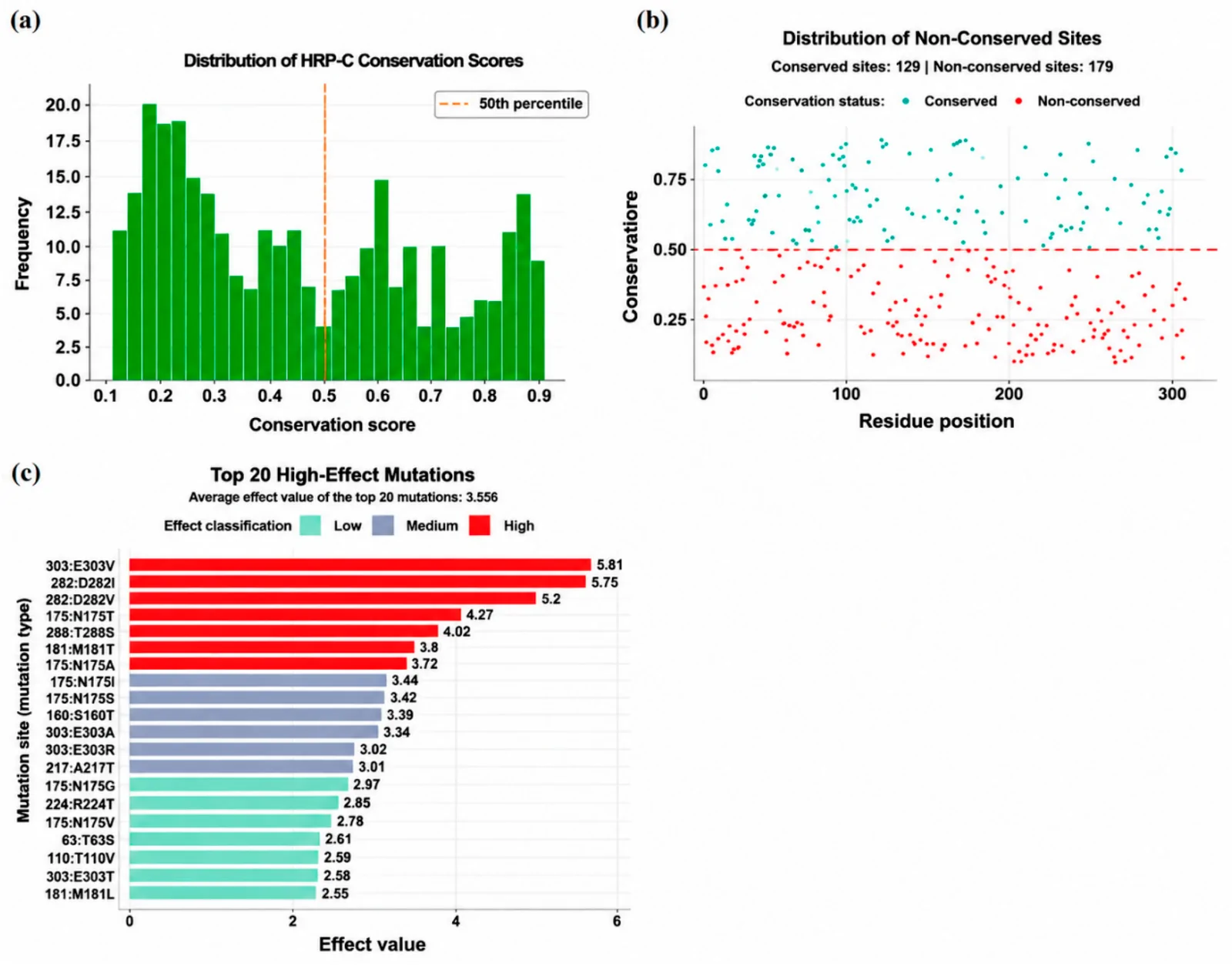
Conservation-based identification and computational prediction of key HRP-C mutation sites. (**a**) Distribution plot of conservation scores, where the dashed line indicates the median threshold; sites with scores below this value are defined as non-conserved sites. (**b**) Distribution of non-conserved sites (red) mapped onto the protein sequence and three-dimensional structure. (**c**) Distribution of virtual saturation mutation scan scores and candidate mutation screening. Mutation sites were classified into three groups based on their predicted effect sizes: high-effect (red), medium-effect (blue), and low-effect (light green).

The potential effects of amino acid substitutions were analyzed using structure-based prediction approaches. Candidate mutations were introduced into the HRP-C sequence, and their potential influence on protein stability was evaluated. According to the computational screening results, several promising mutation sites were selected for construction and expression in *P. pastoris* (Figure 3a–c).

**Figure 4:**
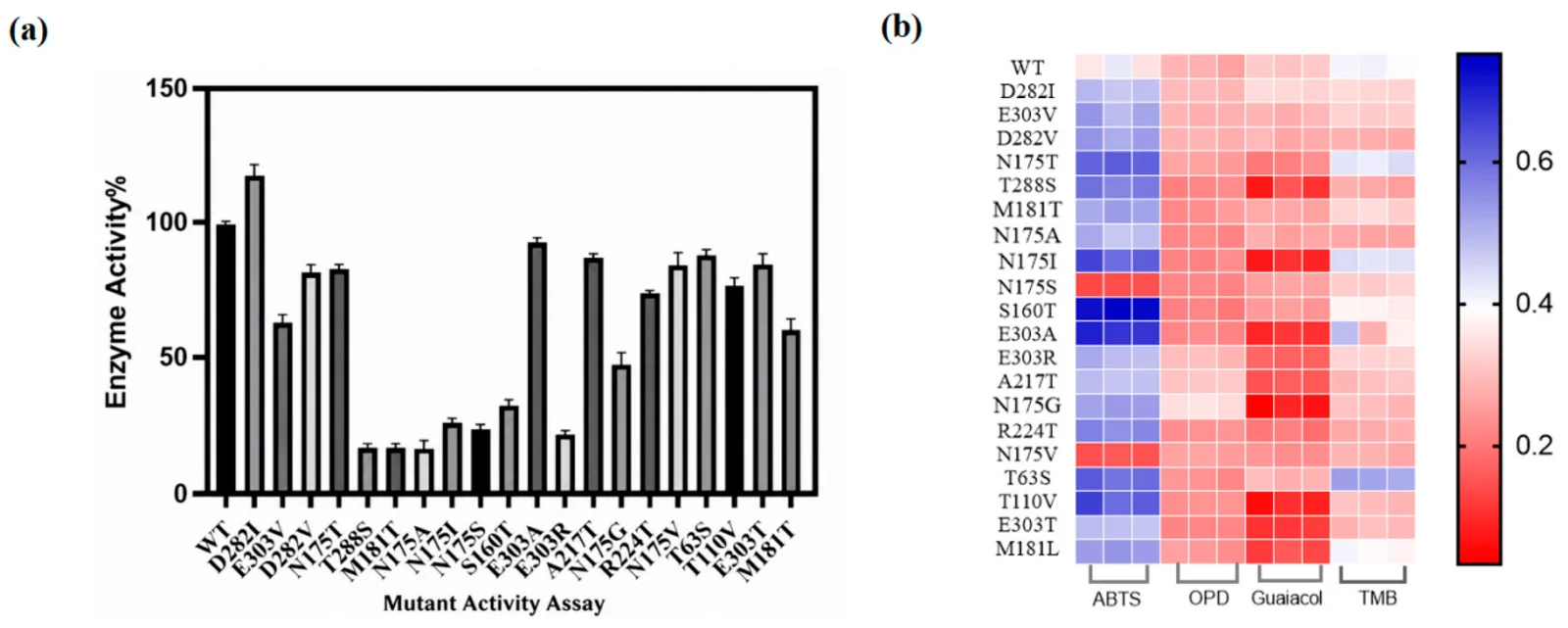
Rational design and functional screening of HRP-C variants. (**a**) Comparison of relative enzyme activity between HRP-C variants and the wild type. (**b**) Comparison of enzymatic activity of different variants on four substrates. Each substrate corresponds to a specific color, with ABTS, o-phenylenediamine(OPD), Guaiacol, and 3,3′,5,5′-tetramethylbenzidine (TMB) each represented by a distinct color in the figure. All experiments were performed in biological triplicate (*n* = 3); data are shown as mean ± standard deviation (SD).

Compared with wild-type (WT) HRP-C, several variants showed improved catalytic performance, among which the D282I variant exhibited the highest increase in specific activity (Figure 4a). The specific activity of HRP-C-D282I was increased by 17.7% compared with the WT enzyme, demonstrating the effectiveness of the rational engineering strategy.

Further substrate specificity analysis showed that different mutations influenced the catalytic preference toward different substrates. Notably, the S160T mutation exhibited improved activity toward ABTS oxidation, whereas D282I maintained enhanced overall catalytic performance (Figure 4b). These results indicated that rational modification of selected residues could effectively regulate HRP-C catalytic properties and provide a practical strategy for developing improved peroxidase variants.

### 2.3. Screening and Functional Characterization of HRP-C Variants

Following the rational identification of potential mutation sites, a series of HRP-C variants were constructed and expressed in *P. pastoris* for functional screening. The catalytic activities of the engineered variants were systematically evaluated and compared with WT HRP-C to identify beneficial mutations that could improve enzyme performance.

For every construct, 24 independent transformants were picked and screened in 24-well plates (504 primary clones in total for the wild type and the 20 variants), and the clones showing a positive colorimetric response after methanol induction were re-screened in shake flasks before quantitative activity comparison.

A total of 20 HRP-C variants were successfully generated and characterized. The activity analysis revealed that different amino acid substitutions resulted in diverse effects on HRP-C catalytic properties, indicating that the selected residues played important roles in regulating enzyme performance. Among these variants, HRP-C-D282I exhibited the most significant improvement in catalytic activity, with a 17.7% increase in specific activity compared with WT HRP-C.

**Figure 5:**
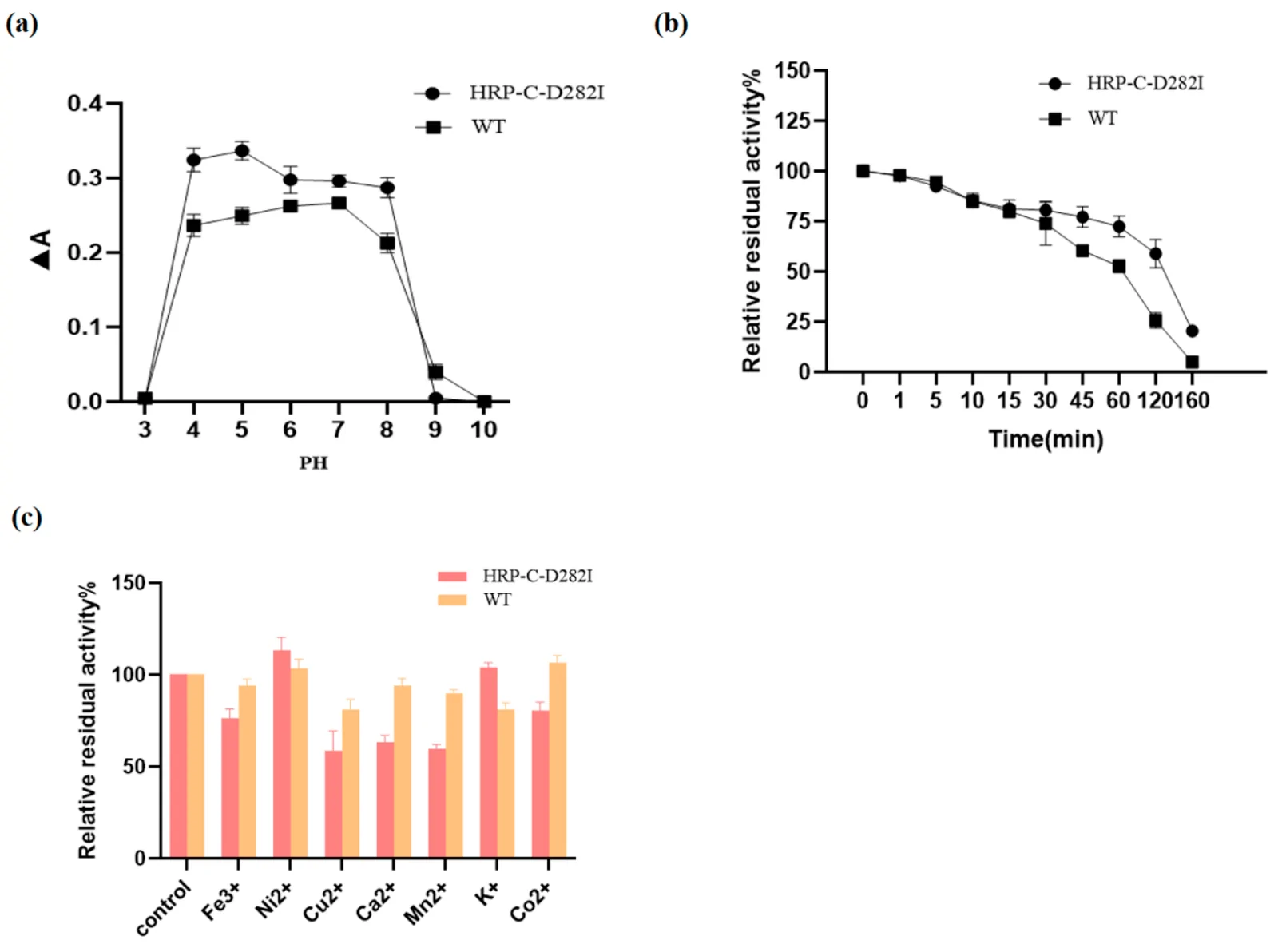
Functional validation and enzymatic characterization of the rationally designed HRP-C-D282I variant. (**a**) pH-activity profile of the D282I variant versus WT HRP-C. Y-axis in (**a**): ΔA, change in absorbance. (**b**) Thermal-stability profiles of the D282I variant versus WT HRP-C at 60 °C. Enzyme activity at 0 min was used for normalization; both raw activity and normalized relative residual activity are provided. Activity-decay data were fitted to a first-order exponential-decay model. Half-life values and their 95% confidence intervals were derived from model fitting. For thermal stability assessment, the purified HRP-C protein was diluted to 0.01 mg/mL as the working stock and incubated at 60 °C for time intervals ranging from 0 to 160 min, and residual enzymatic activity was subsequently measured. All thermal-stability experiments were conducted in biological triplicate (*n* = 3). (**c**) Effects of 1 mM different metal ions on D282I variant and WT HRP-C. The no-metal treatment was used as the control and was defined as 100% relative activity. Metal ion tolerance assays demonstrated that the D282I variant exhibited superior stability compared to the WT against various metal ions. In the presence of Ni^2+^ and K^+^, the residual activities of D282I were 113% and 103% of the control group, respectively, significantly higher than those of the WT (15% and 24%). Relative activities were calculated, and all metal-ion-treatment experiments were conducted in biological triplicate (*n* = 3); data are presented as mean ± standard deviation (SD).

The industrial applicability of HRP-C-D282I was further evaluated by characterizing its biochemical properties under different reaction conditions. Compared with WT, the D282I variant maintained higher activity across a broader pH range, particularly under alkaline conditions (pH 8.0–9.0), suggesting improved environmental adaptability. In addition, HRP-C-D282I exhibited enhanced thermostability at 60 °C. Enzyme activity measured at the 0 min time point was set as 100% for normalization, and both raw absolute activity values and normalized relative residual activity are reported. After 60 min incubation at 60 °C, HRP-C-D282I retained approximately 70% of its initial activity, whereas wild-type HRP-C lost more than 50% of its initial activity under identical conditions (Figure 5b).

The time-course decay data were fitted to a first-order exponential-decay model. The fitted half-life (t_1_/_2_) of HRP-C-D282I was 150 min (95% confidence interval: 136–168 min), which was 2.14-fold longer than the wild-type value of 70 min (95% confidence interval: 62–79 min). Statistical comparison indicated a significant difference in thermostability between the D282I variant and WT HRP-C (*p* < 0.05). All thermostability experiments were performed in biological triplicate (*n* = 3).

The tolerance of HRP-C-D282I toward metal ions was also investigated. The variant showed significantly improved resistance to metal ion inhibition, retaining 113% and 103% of its initial activity in the presence of Ni^2+^ and K^+^, respectively, while WT retained only 15% and 24% activity under the same conditions (Figure 5c). These results indicate that the D282I mutation enhanced the robustness of HRP-C under potentially unfavorable reaction environments.

Furthermore, kinetic analysis demonstrated that the effect of the D282I mutation was substrate-dependent. Compared with WT, HRP-C-D282I showed improved catalytic efficiency (kcat/Km) toward ABTS, OPD, and guaiacol, with increases of 2.28-, 2.03-, and 2.16-fold, respectively. However, the catalytic efficiency toward TMB decreased by 1.81-fold (Table 1).

Overall, the D282I variant exhibited improved catalytic activity, stability, and environmental tolerance, demonstrating its potential for industrial applications. Although the mutation did not universally enhance catalytic efficiency toward all substrates, the improved performance under multiple reaction conditions highlights the effectiveness of rational engineering in developing high-performance HRP-C variants.

### 2.4. Impact of N-glycosylation on HRP-C-D282I Stability and Catalytic Activity

As a eukaryotic expression host, *Pichia pastoris* is capable of introducing post-translational modifications, including *N*-linked glycosylation, which may influence recombinant protein folding, stability, and enzymatic activity. Since HRP-C contains multiple potential glycosylation sites, the association between *N*-glycosylation and enzyme performance was further investigated.

**Figure 6:**
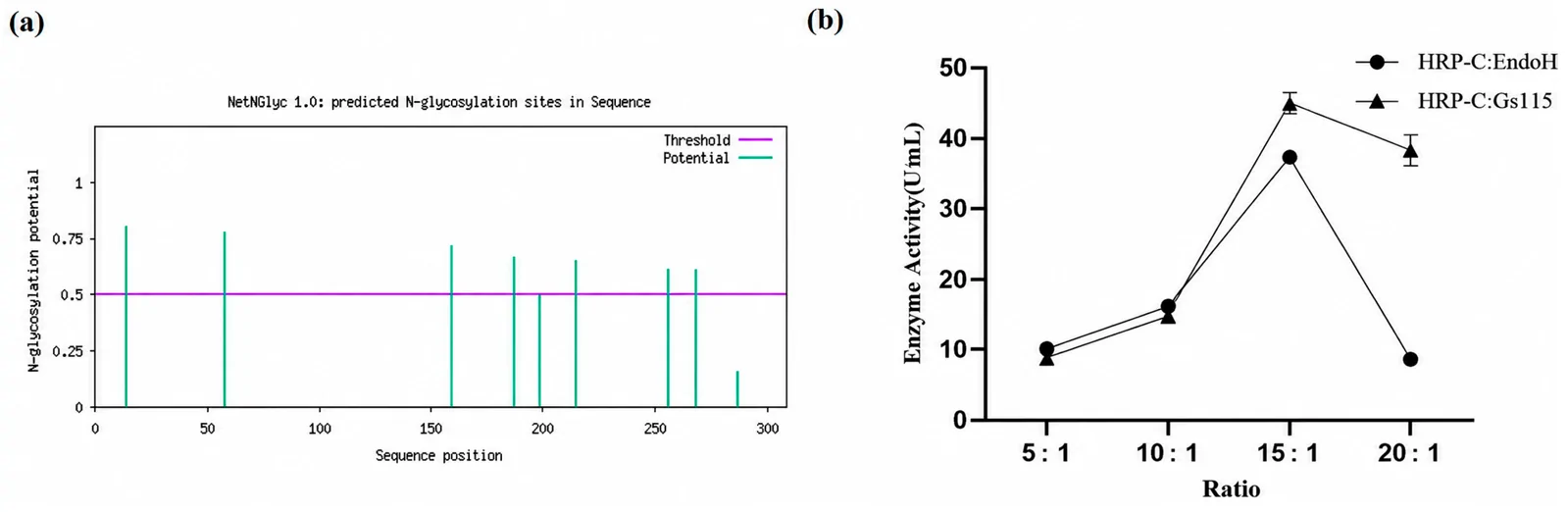
Analysis and functional validation of *N*-glycosylation modification in HRP-C. (**a**) Bioinformatic prediction of putative *N*-glycosylation sites on the HRP-C amino acid sequence. Prediction was performed using the NetNGlyc 1.0 server with a default score threshold of 0.5. Nine high-confidence putative *N*-glycosylation sites were identified: Asn-13, Asn-57, Asn-158, Asn-186, Asn-198, Asn-214, Asn-255, Asn-268, and Asn-286. All candidate sites follow the canonical N-X-S/T sequon (X ≠ Pro) and exhibit prediction scores ≥ 0.5. (**b**) Effect of Endo-H-mediated deglycosylation on HRP-C-D282I enzymatic activity. All experiments were performed in biological triplicate (*n* = 3), and data are presented as mean ± standard deviation (SD).

Bioinformatics analysis identified nine potential *N*-glycosylation sites in HRP-C, including Asn-13, Asn-57, Asn-158, Asn-186, Asn-198, Asn-214, Asn-255, Asn-268, and Asn-286 (Figure 6a). These putative glycosylation sites were distributed throughout the protein sequence, indicating that *N*-linked glycosylation may be associated with the structural and functional properties of recombinant HRP-C.

To investigate the association between *N*-glycosylation and enzyme activity, recombinant HRP-C-D282I was treated with increasing concentrations of endoglycosidase H (Endo H), and the effect of enzymatic deglycosylation on catalytic activity was analyzed. Endo H treatment resulted in a dose-dependent decrease in HRP-C-D282I activity, with greater Endo H treatment being associated with lower residual enzyme activity (Figure 6b).

The reduction in catalytic activity following Endo H treatment suggests that removal of *N*-linked glycans is associated with impaired enzyme performance. Glycosylation may contribute to maintaining the protein conformation required for efficient catalysis; however, the present experiment does not distinguish direct structural effects of glycan removal from other consequences associated *N*-glycosylationwith enzymatic deglycosylation.

Taken together, these results indicate that *N*-linked glycosylation is associated with the functional properties of recombinant HRP-C-D282I under the conditions tested. The findings support a potential contribution of to enzyme performance, but the observed activity decrease following Endo H treatment alone does not establish that *N*-glycosylation is essential for maintaining the structural integrity or catalytic activity of HRP-C-D282I.

### 2.5. Scale-Up Production, Purification, and Lyophilization of HRP-C-D282I

After improving the catalytic performance and stability of HRP-C-D282I through protein engineering, the feasibility of large-scale production was further evaluated. A high-cell-density fermentation process was established in a 50 L bioreactor to investigate the production capability of the engineered enzyme under scalable conditions.

**Figure 7:**
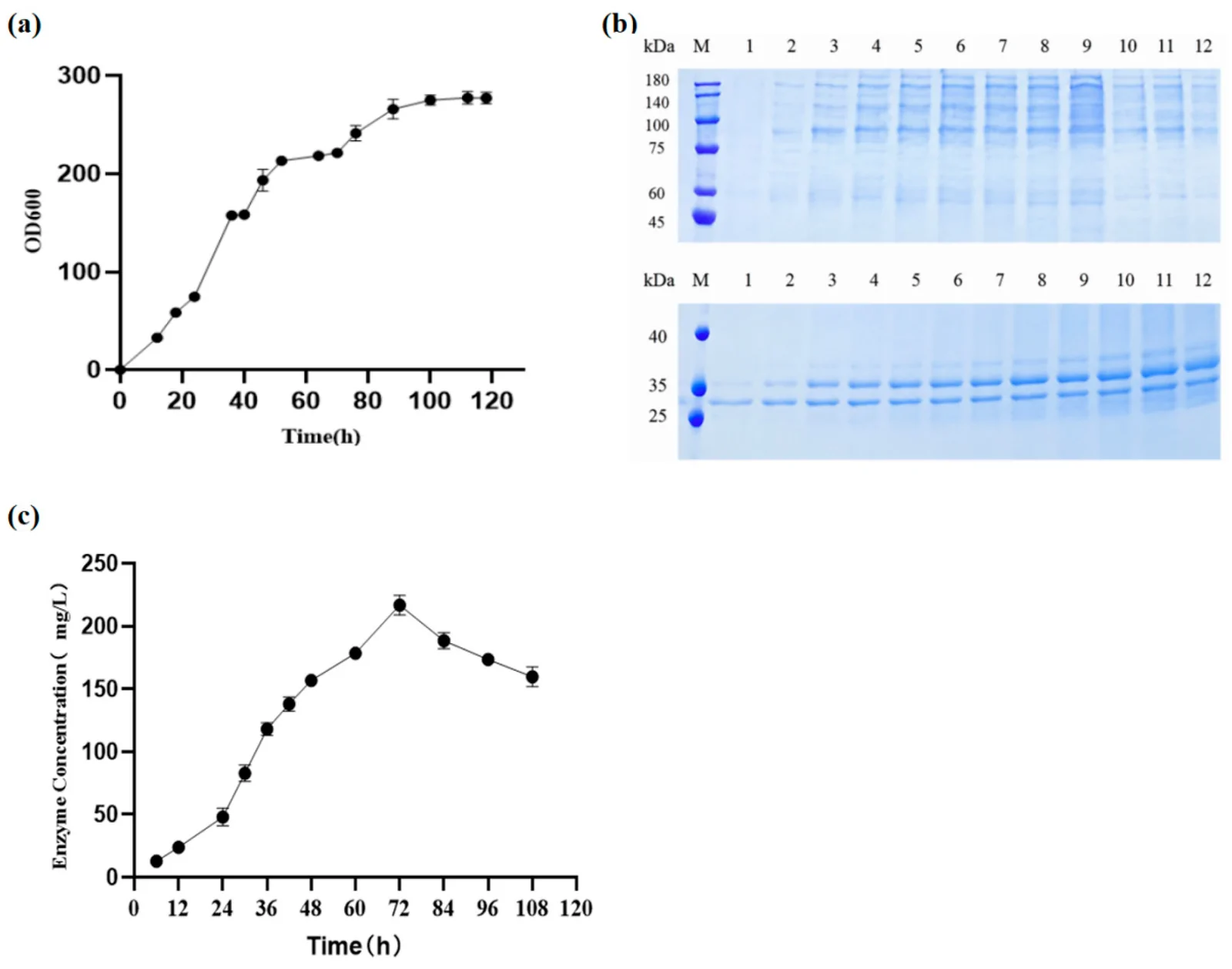
Bioprocess characterization of HRP-C-D282I during high-density fermentation. (**a**) Biomass accumulation profile during the high-density fermentation of HRP-C-D282I. (**b**) SDS-PAGE analysis of HRP-C-D282I expression during fermentation. Lane M: protein molecular weight marker; Lanes 1–12: samples collected at 0 h, 6 h, 12 h, 24 h, 36 h, 48 h, 60 h, 72 h, 84 h, 96 h, 108 h, and 120 h, respectively. Monitoring of the high-density fermentation process revealed distinct kinetic profiles for cell growth (OD_600_) and target protein expression. The cell density increased continuously with fermentation time and plateaued after 96 h, indicating that cell growth had reached saturation. (**c**) High-density fermentation protein concentration curve. The expression level of the target protein accumulated progressively throughout the fermentation process, reaching a maximum protein concentration of 216 mg/L at 72 h.

During the fermentation process, the biomass accumulation and recombinant protein expression profile were monitored throughout cultivation. HRP-C-D282I production gradually increased during the induction phase and reached a maximum concentration of 216 mg/L at 72 h, accompanied by a specific activity of 2108 U/mg (Figure 7a–c). This 50 L bioreactor run serves as a proof-of-concept demonstration, showing that the laboratory-scale shake-flask-derived expression strategy can be transferred to pilot-scale fermentation conditions. Further independent fermentation replicates are still required to systematically evaluate batch-to-batch robustness and true scalability toward industrial manufacturing.

**Figure 8:**
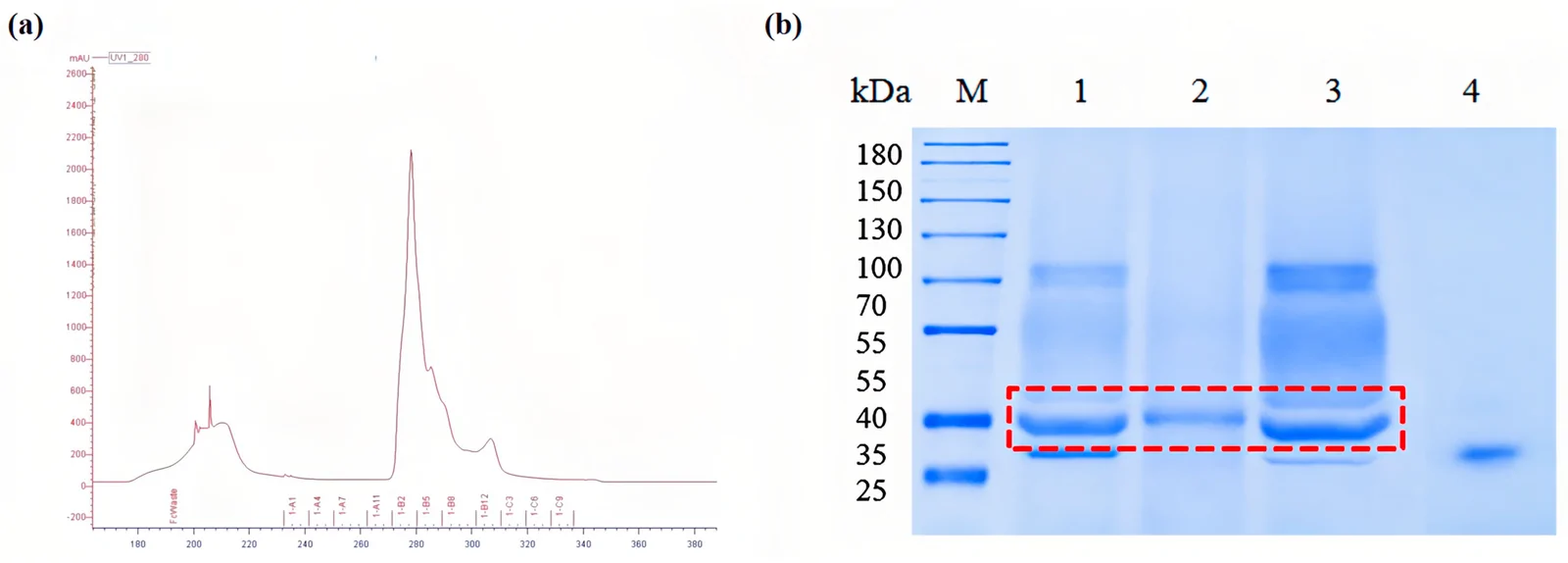
Purification and quality assessment of HRP-C-D282I. (**a**) Chromatogram of the purification process. Peak I: impurities from the flow-through and low-salt wash; Main peak (Peak II): target protein fraction eluted at high salt concentration. (**b**) SDS-PAGE analysis of the purified target protein from the large system. M: protein molecular weight marker; Lanes 1–3: eluted target protein fractions; Lane 4: purified Endo-H enzyme only (independent control sample, not derived from the purification experiment).

To further improve enzyme quality, the fermentation broth was subjected to downstream purification using strong anion-exchange chromatography. The purification process increased the specific activity by 23.4%, from 2108 to 2600 U/mg, while the total activity recovery was 18.85% (Figure 8a,b). The relatively low activity recovery indicates substantial activity loss during downstream processing and highlights the need for further optimization of the purification procedure. Potential losses may originate from non-specific adsorption of HRP-C-D282I onto chromatographic media, enzyme inactivation, and sample-handling-related degradation throughout the purification workflow.

Taking the clarified supernatant of the 4 L purification batch as the reference (864 mg total protein, corresponding to 1.82 × 10^6^ U at 2108 U/mg), the 18.85% activity recovery corresponds to 132 mg of purified enzyme at 2600 U/mg, i.e., a protein recovery of 15.3% and a 1.23-fold increase in specific activity (Table 2).

**Figure 9:**
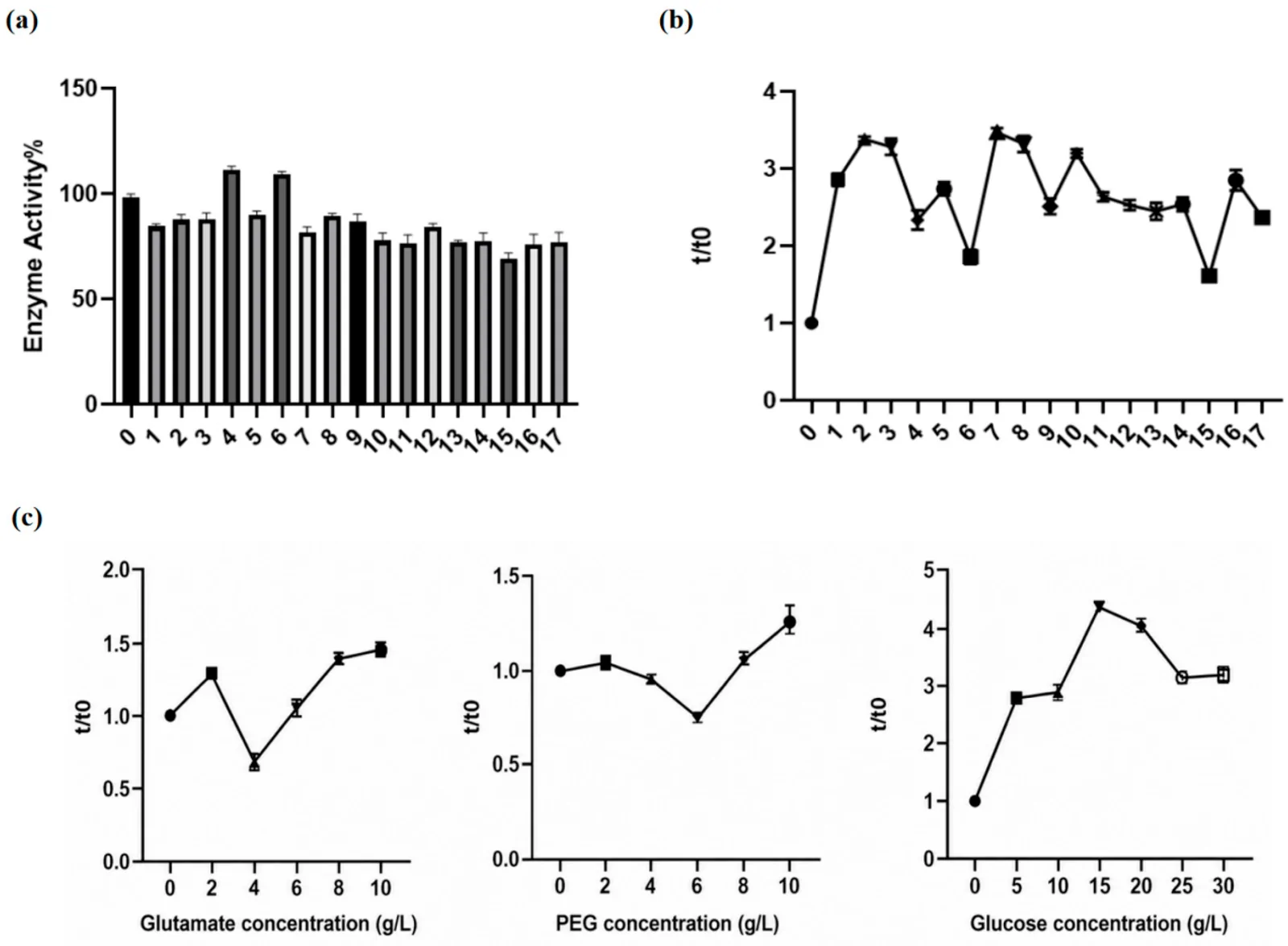
Optimization of lyophilization formulation and evaluation of HRP-C-D282I solid preparation. All experiments were performed in biological triplicate (*n* = 3). (**a**) Determination of enzymatic activity after lyophilization with various protective-agent combinations. (**b**) Relative reconstitution time; the reconstitution time of the lyophilized sample without protective agent (t_0_) was set as reference (t/t_0_ = 1.0), and t_1_/t_0_ represents the normalized reconstitution time for each tested formulation. (**c**) Effects of different concentrations of individual protective agents on normalized reconstitution time of HRP-C-D282I lyophilized powder. All experiments were performed in biological triplicate (*n* = 3), and data are presented as mean ± standard deviation (SD), the graphic symbols in (**b**,**c**) merely visually separate multiple data points, and do not represent the grouping categories.

For long-term storage and practical application, the purified HRP-C-D282I was further formulated into a lyophilized preparation. Sixteen lyoprotectant combinations were systematically screened, determine glucose, glycine and polyethylene glycol as the optimal combination type of protective agents. On this basis, further optimization of component concentrations was carried out, and ultimately the best protective formula was obtained: 5 g/L glucose, 4 g/L glycine and 6 g/L polyethylene glycol, the formula of the experimental group is shown in Table 3. All lyophilization experiments were performed in biological triplicate (*n* = 3). Under the established freeze-drying procedure (−40 °C pre-freezing, staged temperature-ramp drying with a final secondary-drying temperature of 35 °C), the optimized formulation yielded intact lyophilized cakes. The reconstitution time of the lyophilized powder was less than 60 s. The residual moisture content was determined, and the water-loss half-life during lyophilization was calculated to be 15.66 h. We further quantified key functional indices before and after lyophilization. After reconstitution, the lyophilized preparation achieved an enzymatic activity recovery of 92.3% and a protein recovery of 95.1%, with a specific activity of 2580 U/mg (Figure 9a–c). Although the formulation showed favorable short-term performance under laboratory lyophilization conditions, dedicated long-term storage-stability tests are required to fully validate its industrial applicability.

The successful establishment of high-density fermentation, efficient purification, and stable lyophilized formulation provides an integrated production process for recombinant HRP-C-D282I. These results demonstrate the potential of the engineered enzyme for scalable manufacturing and practical industrial applications.

## 3. Materials and Methods

### 3.1. Strains, Plasmids, and Reagents

Horseradish peroxidase C (HRP-C, EC 1.11.1.7) is a class III plant peroxidase naturally derived from horseradish (Armoracia rusticana). The amino acid sequence of HRP-C used in this study was retrieved from the NCBI database. The corresponding coding sequence was optimized according to the codon preferences of *Pichia pastoris* and chemically synthesized by a commercial gene synthesis company. The optimized HRP-C gene was assembled into the pHBM905BDM vector to generate the recombinant expression plasmid pHBM905BDM-HRP-C, which was preserved in our laboratory.

The recombinant plasmid pHBM905BDM-HRP-C was used for heterologous production of HRP-C in *P. pastoris* GS115. According to the plasmid map and sequence analysis, the HRP-C coding sequence is located at positions 1262–2209 bp and is preceded by an *N*-terminal yeast secretion leader sequence, which directs the recombinant protein into the secretory pathway of *P. pastoris*. The construct also contains the *P. pastoris* AOX1 transcriptional termination region (2295–2541 bp) and an AOX1 3′ genomic fragment (7955–8711 bp), which are associated with the yeast expression and genomic integration cassette. The plasmid contains the *E. coli* bla (AmpR) gene for ampicillin selection and a ColE1/pMB1/pBR322/pUC-type origin of replication for plasmid propagation in *E. coli*.

*P. pastoris* GS115 was used as the yeast expression host, while *E. coli* DH5α and XL10-Gold were used for plasmid construction and propagation. The recombinant plasmid pHBM905BDM-HRP-C and the *P. pastoris* GS115 strain were preserved in our laboratory. All restriction enzymes, T5 exonuclease, high-fidelity DNA polymerases, and other molecular biology reagents were purchased from New England Biolabs Inc (Ipswich, MA, USA). Chromatographic media and purification materials were obtained from GE Healthcare (Uppsala, Sweden). Unless otherwise stated, all other chemicals and reagents were of analytical grade or higher.

### 3.2. Construction of HRP-C Variants

The amino acid sequence of HRP-C comprises 308 residues. To identify suitable mutation sites for improving the stability of HRP-C, sequence conservation analysis and structure-based stability prediction were performed. ConSurf (https://consurf.tau.ac.il/, accessed 19 November 2024) analysis was first used to evaluate the evolutionary conservation of the HRP-C sequence. Based on the conservation scores, the lower 50% of the residues were defined as non-conserved positions, resulting in the identification of 179 non-conserved residues.

The 179 non-conserved positions were subsequently subjected to virtual saturation mutagenesis using FoldX (v5.0). For each non-conserved position, all 19 possible amino acid substitutions other than the original residue were evaluated. The predicted change in folding free energy (ΔΔG) was calculated for each substitution to assess its potential effect on protein stability. Based on the predicted stability effects, the 20 substitutions with the most favorable ΔΔG values were selected as candidate mutations for experimental validation.

Site-directed mutagenesis was performed to introduce the selected amino acid substitutions into the HRP-C gene. The corresponding mutant plasmids were constructed using the appropriate mutation primers, and the resulting plasmids were verified by DNA sequencing before transformation into *P. pastoris* GS115 for expression analysis. Detailed information regarding the 20 candidate mutations and the corresponding site-directed mutagenesis primer sequences for the representative plasmids used in the primer construction is presented in Supplementary Table S1 and Supplementary Table S2, respectively.

### 3.3. Transformation and Expression of Recombinant HRP-C Variants

The recombinant plasmids carrying the wild-type HRP-C gene or the corresponding mutant genes were linearized with SalI prior to transformation into *Pichia pastoris* GS115. For electroporation, 100 μL of competent *P. pastoris* GS115 cells were mixed gently with 5–10 μg of purified linearized plasmid DNA and incubated on ice for 2 min. The mixture was then transferred into a pre-chilled 0.2 cm electroporation cuvette and subjected to electroporation at 1500 V, 25 μF, and 200 Ω, with a time constant of 4–5 ms. Immediately after electroporation, 400 μL of pre-chilled sterile 1 M sorbitol and YPD medium were added for recovery. The cells were recovered at 28 °C and 220 rpm for 2 h; the cell suspension was centrifuged at 5000 rpm, 500 μL of the supernatant was discarded, and the remaining cells were spread on MD selection plates and incubated at 28–30 °C for 2–4 days until single transformants appeared, positive transformants were selected and verified by genomic PCR. For each HRP-C construct (the wild type and the 20 variants), 24 independent transformants were picked, and every single colony was inoculated into one well of a 24-well plate containing 4 mL of BMGY, so that the 24 clones of one construct occupied one plate. The plates were covered with sterile breathable sealing film and incubated at 28 °C and 220 rpm for 36–48 h, giving 504 primary clones in total (21 constructs × 24 transformants). Although multiple independent transformants were screened for each construct, gene copy number and integration patterns were not quantitatively determined in the present study. Therefore, potential contributions of copy-number variation to differences in recombinant expression cannot be completely excluded.

For induction, approximately half of the spent medium (2 mL per well) was removed and replaced with an equal volume of BMMY. After glycerol depletion, methanol was added every 12 h to a final concentration of 0.5–1.0% (*v*/*v*) together with 4–10 μM hemin, and induction was continued for 3–5 days. Culture supernatants were collected by centrifugation at 4 °C and 6000 rpm for 10 min and screened for peroxidase activity with the colorimetric guaiacol–H_2_O_2_ assay, in which a positive color reaction indicated secretion of active HRP-C. Because the expression level obtained in 24-well plates was low, every clone giving a positive color reaction was transferred to shake flasks for confirmation: a single colony was grown in 50 mL BMGY to an OD_600_ of 4–6, harvested by centrifugation, resuspended in 25 mL BMMY, and induced for 4–5 days with methanol added to 1% (*v*/*v*) and 5 μM hemin every 24 h. Supernatants from these shake-flask cultures were concentrated and buffer-exchanged by ultrafiltration, quantified by the Bradford assay, and assayed for activity; only variants that reproducibly outperformed the wild type under these conditions were taken forward for purification and detailed characterization.

For recombinant protein expression, positive *P. pastoris* GS115 transformants were cultured in BMGY medium to obtain sufficient biomass. The cells were subsequently transferred to the induction stage, and methanol was used as the carbon source for recombinant HRP-C expression. Methanol induction was performed for 96 h, during which samples were collected at regular intervals to monitor cell growth and HRP-C production. The recombinant HRP-C variants were subsequently harvested for enzymatic activity analysis and further characterization.

### 3.4. High-Density Fermentation of HRP-C-D282I

Recombinant *P. pastoris* GS115 cells harboring the HRP-C-D282I expression cassette were cultivated in a 50 L bioreactor. The fermentation was carried out at a controlled temperature of 28 °C and pH 5.5 (adjusted with ammonia and phosphoric acid). The dissolved oxygen (DO) level was maintained between 20% and 30% of air saturation by cascading agitation and aeration, with aeration at 2 vvm and agitation at 500 rpm.

After the initial batch phase, glycerol was fed to achieve high cell density. The feeding solution consisted of 50% (*w*/*v*) glycerol supplemented with trace elements. Glycerol depletion was determined by a sharp rise in the dissolved oxygen concentration within 2–3 min, indicating the complete consumption of glycerol. When the wet cell weight reached approximately 180–200 g/L, the feeding strategy was switched to methanol induction, and hemin was added to a final concentration of 5 μM to enhance the production of active HRP-C-D282I.

The methanol-feeding procedure was performed as follows: an adaptation phase with full-speed feeding for 30 s followed by a 1 h stop for adaptation; subsequently, methanol was fed at 1 mL/min for 12 h as the transition phase; the feeding rate was increased to 1.67 mL/min from 12 h to 20 h post-induction, and further adjusted to 2.5 mL/min after 20 h post-induction. The methanol feeding rate was dynamically adjusted according to the real-time metabolic status of the culture and was coupled with the dissolved oxygen level, which was maintained within 20–30%. Induction was sustained for 96 h. Samples were collected every 12 h during the induction period to monitor biomass accumulation, recombinant protein production, and enzyme activity.

### 3.5. Purification of Recombinant HRP-C-D282I

The fermentation broth was collected and centrifuged to remove cells and insoluble components. The clarified supernatant was further filtered and concentrated using tangential flow filtration with a 5 kDa molecular weight cutoff membrane.

The concentrated protein solution was buffer-exchanged into 10 mM Tris-HCl (pH 8.0). A 5 mL HiTrap Q HP strong anion-exchange chromatography column was used for small-scale condition screening with a constant flow rate of 1 mL/min. After column equilibration with 10 mM Tris-HCl (pH 8.0), bound proteins were eluted with a linear gradient of 0–1 M NaCl prepared in the same buffer, and the target HRP-C-D282I protein was eluted within the NaCl concentration range of 200–380 mM.

For preparative purification of samples from the 50 L high-density fermentation run, a 385 mL HiTrap Q HP column was applied. The column was equilibrated with 10 mM Tris-HCl (pH 8.0) for 3 column volumes (CV). Sample loading was performed at a flow rate of 17 mL/min. Following sample loading, the column was washed with 6 CV of 10 mM Tris-HCl (pH 8.0). Bound proteins were subsequently eluted at 20 mL/min using a linear 0–1 M NaCl gradient prepared in 10 mM Tris-HCl (pH 8.0) over 6 CV. The target HRP-C-D282I protein was eluted within the NaCl concentration range of 200–380 mM.

Fractions exhibiting high peroxidase activity were collected, pooled, and concentrated for subsequent analysis. Purification quality was verified by SDS-PAGE. The purified HRP-C-D282I samples were analyzed by SDS-PAGE, protein concentration determination, and enzymatic activity assays.

### 3.6. Lyophilization of HRP-C-D282I

Purified HRP-C-D282I was mixed with different lyoprotectant candidates to screen an optimal lyophilized formulation. In total, 16 different lyoprotectant combinations were evaluated. The final optimized formulation contained 5 g/L glucose, 4 g/L glycine, and 6 g/L polyethylene glycol.

For lyophilization, the formulated enzyme solution was sterile-filtered and aliquoted into sterile vials. Samples were pre-frozen at −40 °C, followed by a two-stage freeze-drying process under vacuum: primary drying with gradual temperature elevation from −40 °C to 0 °C, and secondary drying with further gradual heating up to a final temperature of 35 °C. All lyophilization-related experiments were performed in biological triplicate (*n* = 3).

After lyophilization, the lyophilized cake appearance was visually inspected. Reconstitution time was recorded after adding an equal volume of reconstitution buffer. Residual moisture content was determined, and the water loss half-life was calculated as 15.66 h. To assess enzyme performance before and after lyophilization, enzyme activity, total protein concentration, and specific activity were measured for both the starting enzyme solution and reconstituted lyophilized powder. The enzymatic activity recovery and protein recovery after lyophilization were calculated accordingly. Activity recovery was calculated as the ratio of total enzymatic activity after reconstitution to that before lyophilization. Protein recovery was calculated as the ratio of total soluble protein after reconstitution to that before lyophilization. All measurements were performed in biological triplicate (*n* = 3).

### 3.7. Enzyme Activity Assay and Biochemical Characterization

HRP-C-D282I enzymatic activity was determined using the guaiacol oxidation assay according to the national standard GB/T 32131-2015 [25]. One unit (U) of enzyme activity was defined as the amount of enzyme required to catalyze the conversion of 1 μmol substrate per minute under defined assay conditions. Enzyme activity was calculated according to the Beer–Lambert law as follows:

$$Activity\;\left(U/\mathrm{mL}\right)\;=\;\frac{\Delta A\times V_{total}}{\varepsilon \times l\times V_{enzyme}\times t}$$

where  ΔA  is absorbance change per minute (ΔA/min), obtained from the linear initial phase of kinetic progress curves. $V_{total}$ is the total volume of the reaction mixture, 3 mL. ε is molar extinction coefficient of chromogenic substrate (mM^−1^·cm^−1^), as listed in Table 4. l is the optical path length of the quartz cuvette, 1 cm. $V_{enzyme}$ is the volume of added purified enzyme solution, 0.02 mL (20 μL). t is the reaction time, 1 min, corresponding to the unit of ΔA/min.

For the standard activity assay, the total reaction volume was 3 mL. The reaction system contained 1 mM H_2_O_2_, appropriate concentrations of substrate, and enzyme solution in 0.1 M phosphate buffer (pH 7.0). Reactions were performed at 37 °C, and absorbance changes were monitored at the corresponding characteristic wavelength for each substrate. Detailed detection wavelengths and molar extinction coefficients for the four substrates (ABTS, TMB, OPD, and guaiacol) are summarized in Table 4. Blank reactions without enzyme were run in parallel for background correction. The optical path length was 1 cm for quartz-cuvette-based assays, while the typical path length of 96-well microplates for high-throughput screening ranged from 0.5 to 0.7 cm.

For high-throughput substrate-preference screening of HRP-C variants, each reaction was incubated for 2 min and subsequently terminated, and endpoint absorbance was recorded at the corresponding wavelength. These raw endpoint absorbance values were directly employed to generate the heatmap shown in Figure 4b without further conversion to specific activity. The buffer and reagent concentrations for this screening assay were kept consistent with those used for the standard kinetic measurements.

For biochemical characterization of pH and thermal stability, residual enzymatic activity was measured under uniform standard assay conditions. For pH-activity profiling, assays were carried out across a series of pH values using appropriate buffer systems. For pH-stability assays, enzyme samples were pre-incubated at different pH values, followed by activity measurement under standard assay conditions.

For thermal-stability evaluation, purified HRP-C protein (wild-type and D282I variant) was diluted to a working concentration of 0.01 mg/mL and incubated at 60 °C for defined time intervals ranging from 0 to 160 min. The 0 min sample (without heat incubation) served as the reference for normalization. Both raw absolute activity and normalized relative residual activity were recorded. Residual activity was determined immediately after heat treatment. The time-dependent activity-decay curves were fitted by non-linear regression to a first-order exponential-decay model using GraphPad Prism (v10.1.2). The half-life (t_1_/_2_) together with its 95% confidence interval (CI) was calculated from the fitted model. Statistical comparison of half-life parameters between wild-type and D282I was performed, and the corresponding *p*-value was reported. All stability experiments were performed in biological triplicate (*n* = 3).

To evaluate metal-ion tolerance, HRP-C-D282I and wild-type HRP-C were pre-incubated individually with different metal-ion solutions (Fe^3+^, Ni^2+^, Cu^2+^, Ca^2+^, Mn^2+^, K^+^, Co^2+^) at a final concentration of 1 mM prior to the activity assay. The assay without added metal ions served as the control group. Relative residual activity was calculated by setting the activity of the control group to 100%. All metal-ion experiments were conducted in biological triplicate (*n* = 3).

For steady-state kinetic analysis, four representative substrates, including ABTS, TMB, OPD, and guaiacol, were tested. The enzyme working concentration was fixed at 0.01 mg/mL, and 20 μL of diluted enzyme solution was added into each 3 mL total-volume reaction system. The concentration of H_2_O_2_ was kept constant at 1 mM. Substrates were assayed over a concentration gradient ranging from 0.05 mmol/L to 10 mmol/L. All kinetic measurements were performed in biological triplicate (*n* = 3). Initial reaction rates were recorded at 37 °C. The kinetic parameters (Km, Vmax, and kcat) were obtained by non-linear regression fitting of the initial-rate data to the Michaelis–Menten equation using GraphPad Prism.

### 3.8. Glycosylation Analysis of HRP-C-D282I

To predict potential *N*-glycosylation sites, the amino-acid sequence of HRP-C was analyzed using the NetNGlyc 1.0 server with the default threshold value of 0.5. Candidate *N*-glycosylation sites were screened according to two criteria: a NetNGlyc prediction score ≥0.5, and compliance with the canonical N-X-S/T sequon (where X represents any amino-acid residue except proline). Nine putative *N*-glycosylation sites were identified, located at Asn13, Asn57, Asn158, Asn186, Asn198, Asn214, Asn255, Asn268, and Asn286.

For experimental deglycosylation assays, purified recombinant HRP-C-D282I was treated with a gradient of Endo-H glycosidase concentrations to remove *N*-linked glycans. After deglycosylation treatment, changes in molecular weight were assessed by SDS-PAGE. The guaiacol oxidation assay was further applied to measure the residual enzymatic activity of Endo-H-treated samples.

It should be noted that the reduction in enzyme activity after Endo-H-mediated deglycosylation alone cannot fully prove that *N*-glycosylation is absolutely essential for maintaining the structural integrity and catalytic activity of HRP-C. Additional orthogonal evidence such as site-directed mutagenesis of individual glycosylation-site residues, circular dichroism-based secondary-structure monitoring, or thermal-shift-assay-based protein-stability evaluation would be required for definitive confirmation. In the present study, the observed activity decrease upon Endo-H treatment only provides correlative evidence indicating that *N*-glycan removal is associated with impaired enzyme performance.

### 3.9. Statistical Analysis

Unless otherwise stated, quantitative experiments were performed using three independent biological replicates (*n* = 3). Biological replicates refer to independently prepared cultures or enzyme samples. Quantitative data are presented as mean ± standard deviation (SD), and error bars represent the SD of the biological replicates. Statistical significance was evaluated using appropriate statistical tests based on the experimental design, with a two-sided *p* value < 0.05 considered statistically significant.

For enzyme stability analysis, time-dependent activity-decay data were fitted to a first-order exponential-decay model using GraphPad Prism, and half-life values (t1/2) with 95% confidence intervals (CIs) were obtained from the fitted models. For steady-state kinetic analysis, K_m_, V_max_, and kcat values were determined by nonlinear regression fitting of the initial-rate data to the Michaelis–Menten equation using GraphPad Prism. Statistical comparisons between experimental groups were performed as appropriate for the corresponding experimental design.

## 4. Conclusions

In this study, a comprehensive engineering and bioprocess optimization strategy was developed to improve the catalytic performance and production efficiency of recombinant HRP-C. By combining hemin supplementation, rational protein engineering, and systematic functional evaluation, an enhanced HRP-C variant with improved catalytic properties was successfully obtained.

Hemin supplementation significantly promoted the formation of active HRP-C, indicating that sufficient heme availability is an important factor for improving the production of functional recombinant peroxidase. Through sequence analysis, computational prediction, and experimental screening, the D282I variant was identified as the most promising candidate among the engineered HRP-C variants. Compared with WT HRP-C, HRP-C-D282I exhibited enhanced catalytic activity, improved pH adaptability, higher thermostability, and increased tolerance toward inhibitory metal ions. Kinetic analysis further demonstrated that the effect of the D282I substitution was substrate-dependent, with improved catalytic efficiency toward several representative substrates.

Furthermore, *N*-glycosylation analysis showed that Endo H-mediated removal of *N*-linked glycans was associated with reduced enzymatic activity of recombinant HRP-C-D282I. These findings support a contribution of *N*-glycosylation to enzyme performance in the *P. pastoris* expression system, while the present data do not establish that glycosylation is essential for structural integrity or catalytic activity.

In addition, the production process of HRP-C-D282I was successfully transferred to a 50 L high-cell-density fermentation system, achieving a maximum enzyme concentration of 216 mg/L with a specific activity of 2108 U/mg. Subsequent purification increased the specific activity to 2600 U/mg, and an optimized lyophilization process further improved the feasibility of enzyme storage and application.

Overall, this study provides an effective strategy for developing high-performance recombinant peroxidases through the integration of molecular engineering and process optimization. The engineered HRP-C-D282I variant, together with its scalable production and stabilization process, demonstrates promising laboratory-scale performance for future industrial translation.

## Figures and Tables

**Table 1:** Enzymatic kinetic parameters of HRP-C wild-type and D282I variant.

Substrate	Enzyme	Km (μM)	Vmax (μM/min)	kcat (s^−1^)	kcat/Km (μM^−1^s^−1^)
ABTS	D282I	0.1341 ± 0.015	0.1957 ± 0.004	98	730
	WT	0.1810 ± 0.033	0.1175 ± 0.004	58	320
TMB	D282I	0.3447 ± 0.081	0.6367 ± 0.029	318	922
	WT	0.1217 ± 0.025	0.4070 ± 0.015	203	1668
OPD	D282I	0.1087 ± 0.018	0.4374 ± 0.012	218	2005
	WT	0.2562 ± 0.067	0.5077 ± 0.024	253	987
Guaiacol	D282I	0.8991 ± 0.103	0.4195 ± 0.013	210	233
	WT	1.323 ± 0.304	0.2871 ± 0.020	143	108

**Table 2:** Purification of recombinant HRP-C-D282I from a representative 4 L batch of the 50 L high-cell-density fermentation.

Purification Step	Volume (mL)	Total Protein (mg)	Total Activity (U)	Specific Activity (U/mg)	Activity Recovery (%)	Protein Recovery (%)	Purification Fold
Clarified fermentation supernatant	4000	864	1.82 × 10^6^	2108	100	100	1.00
TFF concentration and buffer exchange (5 kDa MWCO; 10 mM Tris-HCl, pH 8.0)	800	838	1.75 × 10^6^	2088	96.2	97.0	0.99
Strong anion-exchange eluate(385 mL HiTrap Q HP; 200–380 mM NaCl)	600	136	3.55 × 10^5^	2610	19.5	15.8	1.24
Final concentrated eluate (5 kDa ultrafiltration, final product)	200	132	3.43 × 10^5^	2600	18.85	15.3	1.23

**Table 3:** Lyoprotectant Formulations for Different Experimental Groups.

Group	Formula (g/L)	Group	Formula (g/L)
1	Sucrose 20, Histidine 5, Polyethylene glycol 5	10	Sucrose 20, Glycine 5, Albumin 5
2	Sucrose 20, Glycine 5, Polyethylene glycol 5	11	Mannitol 20, Glycine 5, Albumin 5
3	Trehalose 20, Histidine 5, Polyethylene glycol 5	12	Trehalose 20, Glycine 5, Albumin 5
4	Trehalose 20, Glycine 5, Polyethylene glycol 5	13	Glucose 20, Histidine 5, Albumin 5
5	Glucose 20, Histidine 5, Polyethylene glycol 5	14	Sucrose 20, Histidine 5, Albumin 5
6	Glucose 20, Glycine 5, Polyethylene glycol 5	15	Mannitol 20, Histidine 5, Albumin 5
7	Mannitol 20, Histidine 5, Polyethylene glycol 5	16	Trehalose 20, Histidine 5, Albumin 5
8	Mannitol 20, Glycine 5, Polyethylene glycol 5	17	Sucrose 20, Histidine 5
9	Glucose 20, Glycine 5, Albumin 5		

**Table 4:** Chromogenic substrates and spectrophotometric parameters used for HRP-C activity determination.

Substrate	Detection Wavelength (nm)	Molar Extinction Coefficient ε (M^−1^·cm^−1^)	ε (mM^−1^·cm^−1^)
ABTS	405	36,000	36.0
TMB	450	59,000	59.0
OPD	492	13,500	13.5
Guaiacol	470	26,600	26.6

## Data Availability

The original contributions presented in this study are included in the article. Further inquiries can be directed to the corresponding authors.

## Supplementary Materials

The following supporting information can be downloaded at: https://www.mdpi.com/article/10.3390/molecules31203730/s1. Supplementary Table S1: The top 20 candidate mutations in the FoldX virtual saturation mutagenesis analysis. Supplementary Table S2. Primers used for constructing representative plasmids.
