# Supplementary material for: Rational Engineering and Preparation of Horseradish Peroxidase C
Source: Molecules. 2026 Oct 17;31(20):3730. doi: 10.3390/molecules31203730 (PMC13707735; doi:10.3390/molecules31203730)
Supplement: Supplementary file 1 [file molecules-31-03730-s001.zip › molecules-4558816-supplementary.pdf]

Supplementary Materials

Supplementary Table S1. The top 20 candidate mutations in the FoldX virtual saturation mutagenesis analysis.

SerialNumber	Mutation	Serial Number	Mutation
1	E303V	11	E303A
2	D282I	12	E303R
3	D282V	13	A217T
4	N175T	14	N175G
5	T288S	15	R224T
6	M181T	16	N175V
7	N175A	17	T63S
8	N175I	18	T110V
9	N175S	19	E303T
10	S160T	20	M181L

Supplementary Table S2. Primers used for constructing representative plasmids.

Primer Name	Primer Sequence (5′→3′)
G69P-F	GATGCTTTTCCAAACGCTAATTCTGCTAGAG
G69P-R	GCGTTTGGAAAAGCATCCTTTTCGGTTC
I36L-F	CTGCTTCCTGGTTGAGATTGCATTTTCATGATTG
I36L-R	TCTCAACAAGGAAGCAGCAATTCTTGG
I244L-F	GGGTTTGTTGCAGTCTGATCAAGAATTGTTTTCTTC
I244L-R	CAGACTGCAACAAACCCTTTTGCTCTTCC
K149L-F	CTCAACTTTTGGATTCCTTTAGAAACGTTGGTTTG
K149L-R	GGAATCCAAAAGTTGAGGCAAAGTAAAAAAAGG
K232Q-F	TGATAACCAATACTACGTTAACTTGGAAGAGCAAAAG
K232Q-R	CGTAGTATTGGTTATCAAAAATAGTTGGGGTTCTCTG
M181T-F	GATTTATCACTGACAGATTGTATAACTTCTCTAACACTGG
M181T-R	TCTGTCAGTGATAAATCTACACTGATTCTTACCAAAAG
N175S-F	GGTAAGTCTCAGTGTAGATTTATCATGGACAGATTG
N175S-R	CTACACTGAGACTTACCAAAAGTATGACCACC
T63S-F	CAGATCTGAAAAGGATGCTTTTGGTAACG
T63S-R	CATCCTTTTCAGATCTGAAAGAAGTAGTATTGTCC
